# Chloroplast DNA Phylogeography of Holy Basil (*Ocimum tenuiflorum*) in Indian Subcontinent

**DOI:** 10.1155/2014/847482

**Published:** 2014-01-02

**Authors:** Felix Bast, Pooja Rani, Devendra Meena

**Affiliations:** ^1^Centre for Biosciences, Central University of Punjab, City Campus, Mansa Road, Bathinda, Punjab 151001, India; ^2^Department of Oncology, University of Oxford, Old Road Campus Research Building, Roosevelt Drive, Oxford OX3 7DQ, UK

## Abstract

*Ocimum tenuiflorum* L., holy basil “*Tulsi*”, is an important medicinal plant that is being grown and traditionally revered throughout Indian Subcontinent for thousands of years; however, DNA sequence-based genetic diversity of this aromatic herb is not yet known. In this report, we present our studies on the phylogeography of this species using *trnL-trnF* intergenic spacer of plastid genome as the DNA barcode for isolates from Indian subcontinent. Our pairwise distance analyses indicated that genetic heterogeneity of isolates remained quite low, with overall mean nucleotide *p*-distance of 5 × 10^−4^. However, our sensitive phylogenetic analysis using maximum likelihood framework was able to reveal subtle intraspecific molecular evolution of this species within the subcontinent. All isolates except that from North-Central India formed a distinct phylogenetic clade, notwithstanding low bootstrap support and collapse of the clade in Bayesian Inference. North-Central isolates occupied more basal position compared to other isolates, which is suggestive of its evolutionarily primitive status. Indian isolates formed a monophyletic and well-supported clade within *O. tenuiflorum* clade, which indicates a distinct haplotype. Given the vast geographical area of more than 3 million km^2^ encompassing many exclusive biogeographical and ecological zones, relatively low rate of evolution of this herb at this locus in India is particularly interesting.

## 1. Introduction

*Ocimum tenuiflorum* L. (holy basil), also known as “*Tulsi*,” is an aromatic plant in the basil family Lamiaceae (tribe ocimeae), which is native throughout the eastern world tropics. It is an erect, much branched subshrub, 30–60 cm tall with hairy stems and simple, opposite, green leaves that are strongly scented. Leaves have petioles and are ovate up-to 5 cm long, usually slightly toothed. Recent molecular phylogenetic studies indicate that the tribe ocimeae is originated in tropical Asia and got introduced elsewhere [1]. Tulsi has been used as a medicinal plant for thousands of years in Indian traditional medicine Ayurveda and its allied herbalism disciplines for its diverse healing properties. The plant is considered sacred and is worshipped in a sanctorum of its own in traditional Hindu temples, sacred groves, and households throughout the subcontinent and therefore its taxonomical synonym *O. sanctum *L. is more popular in Indian scientific literature. A number of recent biochemical and physiological studies indicate that this plant has antidiabetic [2], antimicrobial [3], anticancer [4], adaptogenic [5], and radioprotective [6] properties.

Genus *Ocimum *was described in 1753 by Linneaus, who listed five species in it. The *Ocimum* genus is characterized by a great variability of both morphology and chemotypes due to the ease of cross-pollination that leads to a large number of interspecific hybrids, subspecies, varieties, and forms, with varying essential oil compositions and morphological characters [7]. Three sections are currently recognized within this genus, namely, *Ocimum Benth.* (with appendiculate posterior stamens, comprised of *basilicum*, *gratissimum*, and *americanum*), *Hierocymum Benth.* (with fascicles of hairs at the base of posterior stamens, comprised of *tenuiflorum*) and *Gymnocymum Benth.* (with glabrous posterior stamens, comprised of *campechianum*) [8]. *O. tenuiflorum* is reported to have a karyotype of 2*n* = 36, which is the lowest among members of *Ocimum* genus [9].

Although this important medicinal plant is being grown and traditionally revered throughout Indian Subcontinent for thousands of years, genetic diversity of this aromatic herb is not yet known. While information on its medicinal properties is readily available (with more than 400 articles available at NCBI-Pubmed Database), relatively less literature is available regarding the diversity of this plant in the world. There are no clear synapomorphies recognized for *Ocimum* genus and it could well be paraphyletic. Interspecific genetic heterogeneity of wild and domesticated *Ocimum* genus in Brazil conducted using RAPD markers demonstrated that wild varieties had lowest within-species similarity indices [10]. In yet another study using RAPD markers, *O. tenuiflorum* was found to be very closely related with *O. gratissimum *[9]. The same study also concluded that *O. tenuiflorum* was the most divergent species according to genetic distances. There were two previous attempts to categorize PCR marker based interspecific genetic diversity of *Ocimum* species in India. In one study, *O. sanctum* (=*tenuiflorum*) showed highest similarity with *O. gratissimum* [11] while, in the other, *O. sanctum* (=*tenuiflorum*) showed highest similarity with *O. americanum* [12]. Reports on either sequence-based phylogeography or intraspecific genetic heterogeneity of *O. tenuiflorum* are nonexistent, even at international level.

Do the plants commonly recognized in India as Tulsi belong to one species? Is there any distinct biogeographic structure in the distribution of this species in India? These are some of the questions attempted to address in the present investigation. In this study sequence-based genetic diversity of Tulsi from Indian subcontinent is carried out for the first time using plastid encoded *trnL-trnF* intergenic spacer sequences.

## 2. Material and Methods

### 2.1. Taxon Sampling

Geographic isolates of *O. tenuiflorum* plants were collected from locations enlisted in Table 1. No special permission was required for the sampling as none of the locations included in the present study were part of the places designated as protected by the government of India. In order to investigate intrapopulation genetic heterogeneity, four samples from one population were collected. Collected samples were stored in deep freezer (−80°C) till further molecular analysis.

Total genomic DNA was extracted from the frozen specimens using HiPurA Plant Genomic Extraction Kit (HiMedia Laboratories, India). Tissues from the apical part of young Tulsi leaves were selected to increase DNA yield. Vortexing was avoided in all steps to prevent shearing of DNA.

Six microliters of diluted DNA solution (containing 10 ng of genomic DNA) was added to each 25 *μ*L reaction mix containing 2.5 *μ*L of 10x reaction buffer (Imperial Life Sciences, India), 4 *μ*L each of 10 *μ*M primer, 2 *μ*L of 1 *μ*M dNTP mixture containing dATP, TTP, dCTP, and dGTP (Imperial Life Sciences, India), 1 unit of r*Taq* DNA polymerase (Imperial Life Sciences, India), and sterile water. Primers used for amplifying *trnL-trnF* spacer were obtained from Taberlet et al. 1991. Reactions also contained 5% DMSO. PCR amplifications were carried out in programmable thermal cycler (Veriti, ABI, USA) and reaction profile included an initial denaturation at 94°C for 3 minutes, followed by 40 cycles of 94°C for 0.5 minutes, 50°C for 2 minutes, and 72°C for 1.5 minutes, and a final extension of 72°C for 10 minutes. Amplified products and a standard *λ*-DNA Hind-III digest were electrophoresed on 1.5% agarose gels for 30 min at 100 V and visualized with ethidium bromide in order to determine approximate length and purity. Positive reactions were purified using ExoSAP-IT PCR clean-up kit following manufacturer's instructions (USB Corporation, Cleveland, OH, USA). PCR amplification reactions (as well as its sequencing) were carried out in duplicate for each target sequence of each isolate using the same set of primers in order to confirm fidelity of *Taq* polymerase.

### 2.2. DNA Sequencing

Purified PCR products were sequenced using a dideoxy chain termination protocol with ABI BigDye Terminator Cycle Sequencing Ready Reaction Kit v 3.1 (Applied Biosystems, Foster City, CA, USA) and a programmable thermal cycler (Veriti, ABI, USA). Two reactions were used to amplify both strands (i.e., one with forward primer and the other with reverse primer). In order to eliminate unincorporated dye terminators, SDS (0.2% final concentration) was added to the cycle sequencing reaction products and heat treated at 98°C for 5 minutes, followed by 25°C for 10 minutes. Reactions were then purified by Centri-Sep spin column (Applied Biosystems, Foster City, CA, USA). Purified extension products were vacuum dried and DNA sequencing was performed (Applied Biosystems 3730*xl* Genetic Analyzer, Foster City, CA, USA). DNA sequences were captured as color-coded electropherograms and were assembled using computer program CodonCodeAligner (CodonCode Corporation, USA). Original sequences are available from the first author upon request and had been submitted to Genbank, with accession numbers as provided in Table 1.

### 2.3. Multiple Alignment and Phylogenetic Analysis

Alignment included additional 2 sequences of related taxa procured from GenBank (Table 1). Sequences were first aligned by MUSCLE algorithm and alignments were edited manually. The ends of aligned sequences were trimmed to minimize the number of missing sites across taxa. Best-fitting nucleotide substitution models were tested using ML ModelTest in MEGA. The model with lowest Bayesian Information Criterion (BIC) score was Tamura-2-Parameter model [13], with BIC score of 2421.106. Pairwise distances between sequences were calculated using *p*-distance model and Tamura-2-Parameter model in MEGA (http://www.megasoftware.net/). Positions containing gaps and missing data were completely eliminated.

Phylogenetic analysis using maximum likelihood (ML) algorithm was conducted using PhyML plug-in v 2.4.5 [14] inside computer program Geneious Pro v 6 (available at http://www.genious.com/) with starting tree generated by BioNJ. Substitution bias was modelled by the Tamura-2-Parameter model. Heuristic searches were performed with tree bisection-reconnection, MULTREES, and steepest descent options in effect. 1000 bootstrap replicates were performed under ML criterion to estimate interior branch support [15]. Bayesian inference (BI) was conducted using MrBayes plug-in v 3 [16] inside computer program Geneious v 6. Analyses were run with four Markov chains for 10^6^ generations with a tree saved every 100th generation. First 1000 trees were discarded as burn-in. A consensus tree was then constructed using the consensus tree builder within Geneious.

## 3. Results

A total of thirteen sequences were generated for *trnL-trnF* spacer region of *O. tenuiflorum* Indian Isolates (listed in Table 1), all of which showed homology with the only available sequence of this species at this locus in Genbank, Accession Number AJ505473 [1]. Length of the *trnL-trnF* spacer region of annotated sequences ranged between 846 bp and 848 bp. Total length of the final sequence alignment was 891, including gaps. All four sequences from one population in Bathinda, Punjab—that was done to assess intrapopulational genetic heterogeneity—were 100% identical and therefore only one among these was included in our subsequent analyses.

Results of pairwise distance analysis (Table 2) indicated comparatively low rate of nucleic acid substitution at this locus. Distances ranged between 0.000 and 0.002 in both of the analyses using *p*-distance and Tamura-3-parameter substitution models. Overall mean distance of the dataset, calculated using either nucleotide *p*-distance or Tamura-3-parameter, was 5 × 10^−4^.

**Figure 1:**
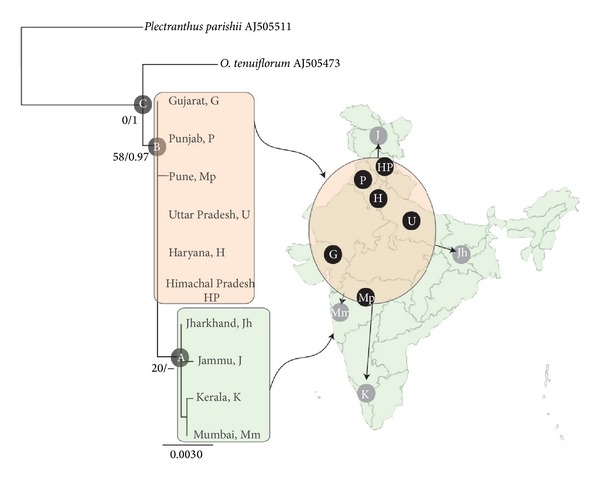
Maximum likelihood (ML) phylogram based on cpDNA *trnL-trnF* spacer sequences, rooted with *Plectranthus parishii* as outgroup. Numbers near nodes represent ML bootstrap proportions exceeding 50, followed by Bayesian Posterior Probabilities. *LnL* = −1405.95401. Scale bar is on the unit of average nucleotide substitutions per site. A map indicating sampling locations of isolates labelled with abbreviations used in the tree is provided on the right, which is appropriately highlighted to mark respective phylogenetic clade/haplotypes. Straight arrows represent dispersal routes from geographical origin. See text for details.

Analyses using maximum likelihood (ML, Figure 1) and Bayesian inference (BI, not given) yielded well-resolved phylograms with comparable topologies. The only difference was presence of an internal clade within Indian isolates (Clade A) in ML. This clade consisted of isolates from elsewhere in India except North-Central region although statistical support for this clade was quite low. Both the analyses resulted in monophyletic clades of Indian isolates (Clade B) as well as all accessions of* O. tenuiflorum* (Clade C).

## 4. Discussion

Our principal finding is that rate of molecular evolution at plastid DNA *trnL-trnF* spacer locus for *O. tenuiflorum* in India is very low but at detectable levels. Given the vast geographical area of more than 3 million km^2^ encompassing many exclusive biogeographical and ecological zones, relatively low rate of evolution of this herb at this locus in India is particularly interesting. Similar low rate of evolution of plastid DNA had been reported for the forest herb *Carex pilosa* in Europe in which no variability was found over 2180 bp sequence throughout its entire distribution area, albeit the area being much smaller than our study [17]. However, a number of phylogeographical studies ascertain the use of plastid DNA to resolve fine structures of spatial distribution of herbaceous plants including below species levels [18–21]. Few studies on the variation of chloroplast genome at intraspecific levels have also been conducted on Indian plants, including *Citrus* [22], *Ceropegia* [23], and Indian Gooseberries [24]. Most of these studies suggest that crucial geological events, such as glaciation, have profound impact on shaping chloroplast genome evolution. Low rate of evolution at this locus observed in this study may be linked to several factors, including type of recolonization process, the number of refugia, or biological features of this species. While Tulsi is grown in temple gardens and households, human intervention in the spread of this species is not to an extent to cause continuous mixing of gene pools.

Phylogenetic reconstruction using ML method has resolved an interesting but subtle clade of isolates from all other parts except North-Central India. This clade was also formed in our distance-based phylogeny reconstruction trials using Neighbour-Joining method (results not given). Bootstrap support for this clade was low and it collapsed in more robust analysis with Bayesian inference. This could be suggestive of a haplotype variant or long-branch attraction—an inherent problem with phylogenetic inference [25]. On the other hand, a basal position of isolates from North-Central India apparent in our analyses indicates that isolates from this region are more “primitive” in evolutionary context. Primitive state of these isolates implies that in ancestral state reconstruction, these taxa would be the one that determines geographical origin of this species. Future studies with extensive taxon sampling and multilocal phylogeny reconstruction are expected to resolve this inference. Isolates from India formed a well-supported clade that is distinct from English isolate in all analyses. This most probably suggests the existence of an Indian haplotype with distinct evolutionary legacy. An interesting arena for prospective research would be to generate sequence data at this locus for isolates from elsewhere in the world, to test theories on the origin of this species and its routes of dispersal.

## 5. Conclusions

The present study revealed for the first time sequence-based phylogeography and molecular evolution of *O. tenuiflorum *in Indian subcontinent. Our results indicate rate of molecular evolution of this species at *TrnL-F* locus remains very low, but at detectable levels. Results also suggest North-Central India as the geographical origin of this species, as indicated by the basal position of North-Central isolates in our ML phylogram. A distinct haplotype of Indian isolates was also revealed in our analyses. Further phylogeographical studies with extensive taxon sampling and other genetic loci are warranted for testing theories of dispersal routes of this important species.

## Figures and Tables

**Table 1:** DNA sequences generated and used in this study, with other pertinent information. Dash (−) represents data unavailable.

Sr. no.	Genbank accession	Location	Latitude (N)	Longitude (E)	Sequence length	GC content (%)	Voucher accession
1	KC894780.1	Jammu, Jammu, and Kashmir	32.73°	74.87°	852	35.3	CUP12OT01
2	KC894781.1	Bhiwani, Haryana	28.78°	76.13°	857	36.1	CUP12OT02
3	KC894782.1	Anand, Gujarat	22.55°	72.95°	852	35.2	CUP12OT03
4	KC894783.1	Chirkunda, Jharkhand	23.71°	86.79°	854	36.1	CUP12OT04
5	KC894784.1	Pune, Maharashtra	18.52°	73.84°	868	36.4	CUP12OT05
6	KC894785.1	Mumbai, Maharashtra	18.96°	72.84°	874	36.2	CUP12OT06
7	KC894786.1	Kozhikode, Kerala	11.25°	75.77°	850	35.8	CUP12OT07
8	KC894787.1	Bilaspur, Himachal Pradesh	31.33°	76.75°	874	36.5	CUP12OT08
9	KC894788.1	Bathinda, Punjab	30.20°	74.94°	851	36.0	CUP12OT09
10	KC894789.1	Bathinda, Punjab	30.20°	74.94°	851	35.8	CUP12OT10
11	KC894790.1	Bathinda, Punjab	30.20°	74.94°	849	35.8	CUP12OT11
12	KC894791.1	Bathinda, Punjab	30.20°	74.94°	851	35.8	CUP12OT12
13	KC894792.1	Chandauli, Uttar Pradesh	25.26°	83.26°	853	35.8	CUP12OT13

**Table 2:** Pairwise distance between aligned sequences. Bottom-left part of the matrix is distance calculated using nucleotide *p*-distance and top right using Tamura-3-Parameter model of nucleotide substitution. Top values are highlighted in both the matrices.

	Bathinda	Gujarat	Haryana	Himachal Pradesh	Jammu and Kashmir	Jharkhand	Kerala	Maharashtra	Pune	Uttar Pradesh
Bathinda		0.000	0.000	0.000	0.001	0.000	0.000	0.000	0.001	0.000
Gujarat	0.000		0.000	0.000	0.001	0.000	0.000	0.000	0.001	0.000
Haryana	0.000	0.000		0.000	0.001	0.000	0.000	0.000	0.001	0.000
Himachal Pradesh	0.000	0.000	0.000		0.001	0.000	0.000	0.000	0.001	0.000
Jammu and Kashmir	0.001	0.001	0.001	0.001		0.001	0.001	0.001	**0.002**	0.001
Jharkhand	0.000	0.000	0.000	0.000	0.001		0.000	0.000	0.001	0.000
Kerala	0.000	0.000	0.000	0.000	0.001	0.000		0.000	0.001	0.000
Maharashtra	0.000	0.000	0.000	0.000	0.001	0.000	0.000		0.001	0.000
Pune	0.001	0.001	0.001	0.001	**0.002**	0.001	0.001	0.001		0.001
Uttar Pradesh	0.000	0.000	0.000	0.000	0.001	0.000	0.000	0.000	0.001	
